# Essential updates 2018/2019: Current topics in the surgical treatment of pancreatic ductal adenocarcinoma

**DOI:** 10.1002/ags3.12379

**Published:** 2020-08-09

**Authors:** Keinosuke Ishido, Kenichi Hakamada, Norihisa Kimura, Takuya Miura, Taiichi Wakiya

**Affiliations:** ^1^ Department of Gastroenterological Surgery Hirosaki University Graduate School of Medicine Hirosaki Japan

**Keywords:** CA19‐9, conversion surgery, minimally invasive pancreatectomy, neoadjuvant treatment, resectability criteria

## Abstract

Pancreatic ductal adenocarcinoma (PDAC) is highly malignant. While cancers in other organs have shown clear improvements in 5‐year survival, the 5‐year survival rate of pancreatic cancer is approximately 10%. Early relapse and metastasis are not uncommon, making it difficult to achieve an acceptable prognosis even after complete surgical resection of the pancreas. Studies have been performed on various treatments to improve the prognosis of PDAC, and multidisciplinary approaches including non‐surgical treatments have led to gradual improvement. In the present literature review, we have described the significance of anatomical and biological resectability criteria, the concept of R0 resection in surgical treatment, the feasibility of minimally invasive surgery, the remarkable development of perioperative chemotherapy, the effectiveness of conversion surgery for unresectable PDAC, and ongoing challenges in PDAC treatment. We also provide an essential update on these subjects by focusing on recent trends and topics.

## Graphical abstract

In the present literature review, we have described the significance of anatomical and biological resectability criteria, the concept of R0 resection in surgical treatment, the feasibility of minimally invasive surgery, the remarkable development of perioperative chemotherapy, the effectiveness of conversion surgery for unresectable pancreatic cancer, and ongoing challenges in pancreatic cancer treatment. We also provide an essential update on these subjects by focusing on recent trends and topics.

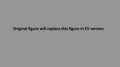

## 1 INTRODUCTION

Pancreatic ductal adenocarcinoma (PDAC) remains an intractable cancer with poor prognosis. The 5‐year survival rates for PDAC are low at approximately 10%.^1^ In many cases, by the time the cancer is detected, during the initial examination, PDAC is diagnosed as unresectable due to advanced local progression or distant metastasis. Currently, PDAC is the fourth most common cause of cancer‐related mortality.^1^ Due to a globally increasing trend,^2^ it is anticipated to become the second leading cause of cancer‐related mortality by 2030,^3^ which would be a major loss to society. However, better treatment outcomes are being noted owing to recent improvements in diagnostic techniques, and advances in multidisciplinary treatment, including surgery, and optimization of surgical indications.^4^ In this present literature review, we aimed to provide an update regarding the development of surgical treatment and multidisciplinary treatment strategies for PDAC.

## 2 SURGICAL TREATMENT

### 2.1 Image‐based resectability criteria

Surgical resection of PDAC is the predominant treatment option, and complete resection (R0 resection) is essential for long‐term survival. Thin‐slice multi‐detector row computed tomography (MDCT) is a standard diagnostic method in cases requiring accurate R0 resection. Not only tumor localization but also degree of proximity to and invasion of the major blood vessels, such as the superior mesenteric artery, the common hepatic artery, the superior mesenteric vein, and the portal vein, are essential for assessing anatomical resectability (resectable, R; borderline resectable, BR; locally advanced unresectable, UR‐LA; and metastatic unresectable, UR‐M).^5^ Treatment algorithms are developed, according to the resectability, based on the National Comprehensive Cancer Network (NCCN) (https://www.nccn.org/professionals/physician_gls/default.aspx#site), the American Society of Clinical Oncology (ASCO),^6^ the European Society of Medical Oncology (ESMO),^7^ and the Japan Pancreas Society (JPS).^8^ Preoperative treatment was recommended for BR patients for whom upfront surgery is associated with a high rate of R1 resection with a poor prognosis. Consequently, neoadjuvant treatment following the aforementioned guidelines was recommended. Even in cases of UR‐LA‐ and UR‐M‐PDAC, it has been determined that resectability should be assessed after chemotherapy or chemoradiotherapy to achieve conversion surgery, which is among the current, more promising, treatment options.

### 2.2 Biomarker‐based resectability criteria

While MDCT evaluation may indicate that the PDAC is resectable, some patients have distant metastases during laparotomy or experience early recurrence postoperatively.^9^ The prognosis for these patients is not promising and if possible, such surgery should be avoided.

#### 2.2.1 Carbohydrate antigen (CA) 19‐9

A high postoperative CA19‐9 level is a well‐established biomarker predicting the prognosis of patients with resected PDAC.^4^, ^10^, ^11^, ^12^, ^13^, ^14^ A high postoperative CA19‐9 level (>37 U/mL) is a risk factor affecting early postoperative recurrence and poor survival.^15^, ^16^, ^17^ Therefore, CA19‐9 is utilized as a diagnostic marker for recurrence during postoperative surveillance.

##### Preoperative CA19‐9 levels in resectable PDAC

Preoperative CA19‐9 level is also known as a risk factor for early postoperative recurrence of R‐PDAC. Therefore, resectability assessment based on the preoperative CA19‐9 levels has recently been proposed to assess potential distant metastases preoperatively.^18^, ^19^ For predicting early postoperative recurrence and poor prognosis based on preoperative CA19‐9 levels, 85 U/mL,^20^ 100 U/mL,^21^ 125 U/mL,^22^ 178 U/mL,^23^ 200 U/mL,^24^ 210 U/mL,^14^ 385 U/mL,^13^ and 500 U/mL^25^ were reported as cut‐off values. Now neoadjuvant treatment can be an option in such R‐PDAC cases with higher CA19‐9 levels.

##### Preoperative CA19‐9 levels after neoadjuvant therapy in patients with BR/UR‐PDAC

A decrease in the CA19‐9 levels after neoadjuvant therapy (NAT) reflects the effect of preoperative treatment; it is a postoperative long‐term prognostic factor and may be a criterion for resectability. Initially, the cut‐off levels of preoperative CA19‐9 levels were set as high as 400 U/mL^26^

or 500 U/mL.^19^, ^27^ Now, most institutes use more strict criteria with lower CA19‐9 levels, as the following preoperative CA19‐9 levels were reported to be indicative of potential metastasis and poor prognosis: 80 U/mL,^28^ 100 U/mL,^29^ 103 U/mL,^30^ 125 U/mL,^22^ 178 U/mL.^23^ In contrast, normalization of the CA19‐9 levels after NAT is an indicator of good long‐term prognosis.^28^, ^31^

##### CA19‐9 before conversion surgery in UR‐PDAC

CA19‐9 level is one of the most useful biomarkers as an adaptation criterion for conversion surgery after neoadjuvant treatment for unresectable PDAC. In many institutions, remarkable reduction or normalization of CA19‐9 level is a mandatory factor to perform conversion surgery. Standard values have been reported as follows: CA19‐9 level < 91.8 U/mL,^32^ <100 U/mL,^29^, ^33^ <150 U/mL,^34^, ^35^ 80% reduction,^36^ 30% reduction.^37^ However, there is no consensus on a standard value.

#### 2.2.2 18F‐fluorodeoxyglucose‐positron emission tomography

18F‐fluorodeoxyglucose‐positron emission tomography (FDG‐PET) may be used to assess the biological aggressiveness of various tumors and predict tumor prognoses. Moreover, in PDAC, a high maximum standardized uptake value (SUVmax) indicates potential distant metastasis. Therefore, it is useful for considering the possibility of distant metastases in patients with resectable PDAC.^31^, ^38^, ^39^, ^40^

#### 2.2.3 Circulating tumor cells

Cancer cells invade the adjacent blood vessels through epithelial‐mesenchymal transition, disseminate through the circulatory system, and metastasize to distant organs. Therefore, circulating tumor cells (CTCs) are reported to predict both potential metastasis and poor prognosis.^41^, ^42^, ^43^ A recent CLUSTER study reported that preoperative CTC counts may predict early recurrence, i.e. up to 12 months after surgery.^44^ Although the origin of CTCs and appropriate detection methods have not been established to date, the dynamics of CTCs reflect the progress of the cancer and responsiveness to treatment; thus, the presence of CTCs may be a potential criterion for resectability.^45^

#### 2.2.4 Other biomarkers

With respect to other biomarkers, the circulating tumor DNA,^46^, ^47^ exosome,^48^ and microRNA^49^ levels have been reported as candidate factors for assessing the biological resectability of PDAC. Nevertheless, they have not been established as resectability criteria to date.

### 2.3 Local radiality and surgical margins

A positive surgical margin in PDAC resection is a strong indicator of poor prognosis, and the distance from the surgical margin to the tumor affects the achievement of complete resection. The prognosis after R0 resection is reported to improve gradually as the distance from the surgical margin gradually increases.^50^, ^51^, ^52^ Therefore, the very definition of the surgical margin is changing. As per the Royal College of Pathologists (RCPath)^53^ and the American Joint Committee on Cancer (AJCC)^54^ guidelines, a distance of at least 1 mm or more between the cancer cell and the resection surface is defined as R0 resection and that of 0‐1 mm is defined as R1 resection; in the Union for International Cancer Control (UICC)^55^ and JPS^56^ guidelines, a different definition of R1 resection is adopted where the distance between the cancer cell and the resection surface is 0 mm.

#### 2.3.1 Rules for the margin distance

There is a marked difference (Table 1) in the R0 resection rate and prognosis noted between cases where resection was performed using the 0‐mm rule and those where it was performed using the 1‐mm rule.^51^, ^52^, ^57^, ^58^, ^59^, ^60^, ^61^, ^62^, ^63^, ^64^, ^65^ Overall, the R0 resection rate is lower for cases where resection was performed using the 1‐mm rule than for those using the 0‐mm rule. In contrast, the median survival time (MST) after R0 resection was prolonged in cases where resection was performed using the 1‐mm rule. Systematic reviews^50^ and meta‐analyses^62^ have also reported that the adoption of the 1‐mm rule both reduced the R0 resection rate and prolonged the overall survival after R0 resection. The optimum cut‐off margin for improving disease prognosis is reported to be ≥ 1.5 mm^50^ and ≥ 2.0 mm.^59^, ^66^ It is, therefore, necessary to specify the margin rule applied when reporting the outcomes of PDAC treatment.

**Table 1:** Comparison of R0 resection rates and survivals between the 0 mm and the 1 mm rule for pancreatic cancer

Author	Year	Period	n	PD/DP/TP	RR, % (0 mm rule)		RR, % (1 mm rule)		MST, mo (0 mm rule)			MST, % (1 mm rule)			
					R0 (0 mm < R)	R1 (R = 0 mm)	R0 (1 mm ≤ R)	R1 (R < 1 mm)	R0 (0 mm < R)	R1 (R = 0 mm)	*P*	R0 (1 mm ≤ R)	R1 (0 < R<1 mm)	R1 (R = 0 mm)	*P*
Kostantinidis^57^	2013	1993‐2008	554	554	72.0	28.0	32.0	68.0	23	14	<.0001	35	16	14	.001
Sugiura^58^	2013	2002‐2010	208	164/42/2	84.0	16.0	65.0	35.0	‐	‐	‐	26	30	23	N/A
Delpero^52^	2017	2008‐2010	147	147/0/0	75.0	25.0	35.0	65.0	32.4	16.7	N/A	53.9	20		N/A
Nitta^51^	2017	1999‐2010	117	117/0/0	81.0	19.0	26.0	74.0	17	12	.0372	20	14		n.s. n.s.
Strobel^60^	2017	2006‐2012	561	561/0/0	41.9	58.1	20.0	80.0	‐	‐	‐	41.6	27.5	23.4	N/A
Hank^61^	2018	2006‐2014	455	0/218/237	46.4	53.6	23.5	76.5	‐	‐	‐	62.4	24.6	17.2	<.0001
Demir^62^	2018	2007‐2014	254	174/44/36	60.2	39.8	42.9	57.1	28.6	16.5	N/A	31.7	17.1		N/A
Ghanch^63^	2019	2000‐2008	1151	‐	68.8	31.2	56.1	43.9	‐	‐	‐	24.9	25.4	18.7	<.0001
Tummers^64^	2019	2006‐2016	322	275/35/12	‐	‐	59.9	40.1	‐	‐	‐	22	15		N/A
Yamamoto^65^	2019	2001‐2015	100	58/37/5	84.0	16.0	43.0	57.0	N/A	N/A	n.s.	N/A	N/A	0.065	

Abbreviations: DP, distal pancreatectomy; mo, months; mo, months; MST, median survival time; n.s., not significant; N/A, not available.; PD, pancreaticoduodenectomy; R, distance from the resection surface to the cancer cell; RR, resection rate; TP, total pancreatectomy.

#### 2.3.2 Surgical margins after neoadjuvant therapy

NAT is expected to improve the curative rate associated with BR/LA‐PDAC by inducing regression of PDAC cells in the vicinity of the major blood vessels. An analysis of data from the National Cancer Database (NCDB) also discovered improved R0 resection rates after NAT.^67^, ^68^ In addition, a meta‐analysis reported that NAT for R/BR‐PDAC resulted in a significant margin‐negative resection and overall survival prolongation. However, margin‐positive resection after NAT is associated with a poor prognosis. It is necessary to maintain an adequate and safe surgical margin even after NAT.^67^, ^68^, ^69^, ^70^

## 3 MINIMALLY INVASIVE SURGERY

For benign pancreatic tumors and low‐grade tumors, short‐term postoperative outcomes of minimally invasive surgery (MIS) are reportedly equivalent to those of open surgery.^71^, ^72^, ^73^, ^74^, ^75^, ^76^, ^77^, ^78^ Conversely, the oncological safety and validity of MIS as surgical treatment for PCDAC are the subject of much discussion.

### 3.1 Laparoscopic distal pancreatectomy

The operative time for laparoscopic distal pancreatectomy (LDP) is longer than that of laparotomy **(**Table 2 **)**; however, LDP is also associated with significantly less blood loss, fewer complications, and shorter duration of hospital stay.^72^, ^73^, ^74^, ^79^ An increasing number of studies have reported on the oncological safety and long‐term prognosis of LDP for PDAC. Table 2 summarizes the previously reported oncologic factors and disease prognosis associated with LDP and open distal pancreatectomy (ODP) for PDAC.^80^, ^81^, ^82^, ^83^, ^84^, ^85^, ^86^, ^87^, ^88^, ^89^, ^90^, ^91^, ^92^, ^93^, ^94^ Propensity score matching (PSM) analysis using data from the NCDB indicated that the R0 resection rate, number of retrieved lymph nodes, and long‐term prognosis were equivalent for LDP and ODP.^95^ In contrast, the PSM analysis in the DIPLOMA study noted a significant difference in the R0 resection rate, postoperative chemotherapy induction rate, and MST, although the number of retrieved lymph nodes was significantly smaller with LDP.^94^ The concerning issue is that both studies reported a high conversion rate of 20%‐30%. In a recent meta‐analysis, the R0 resection rate, postoperative chemotherapy induction rate, and overall survival rate were similar; however, a large allocation bias was noted in the degree of disease progression. Consequently, a definitive conclusion could not be drawn.^79^ In the future, larger randomized controlled trials (RCTs) are required to compare LDP and ODP for PDAC.^71^

**Table 2:** Comparison of oncological outcomes between LDP and ODP for pancreatic cancer

Author	Year	Study design	Country	n		R0 rate, %	*P*	Harvested LNs	*P*	AT rate, %	*P*	MST, mo	P
Kooby^80^	2010	Rter	USA NCDB)	LDP	23	74.0	.98	13.8 ± 8.4	.47	57.0	.23	16	.71
				ODP	189	73.0		12.5 ± 8.5		70.0		16	
Magge^81^	2013	Rter	USA	LDP	28	86.0	>.99	12 (6‐19）	.75	‐	‐	HR 1.11, *P* = .80	
				ODP	34	88.0		11 (8‐20）		‐			
Rehman^82^	2014	Pros	UK	LDP	8	88.0	.794	16 (1‐27）	.53	‐	‐	33	.91
				ODP	14	86.0		14 (0‐26）		‐		52	
Lee^83^	2014	PSM	Korea	LDP	12	70.0	.426	11.7 ± 7.2	.887	70.0.	.765	60	.616
				ODP	78	87.5		12.1 ± 8.1		650		60.72	
Hu^84^	2014	Pros	China	LDP	11	100	‐	14.8 ± 4.5	.875	‐	‐	5ys, 22%	n.s.
				ODP	23	100		16.1 ± 5.7		‐		5ys, 20%	
Sharpe^85^	2015	Retr	USA NCDB)	LDO	144	87.0	.042	14.9 ± 10.0	.085	‐	‐	‐	‐
				ODP	625	78.0		13.3 ± 9.9		‐		‐	
Shin^86^	2015	Retr	Korea	LDP	70	75.7	.22	12 (1‐34)	.13	78.6	.18	33.4	.25
				ODP	80	83.8		10 (1‐64)		68.8		29.1	
Sulpice^87^	2015	Retr	France (FHD)	LDP	347	‐	‐	‐	‐	‐	‐	62.5	<.0001
				ODP	2406	‐		‐		‐		36.7	
Zhang^88^	2015	Retr	China	LDP	17	94.1	.65	9 (5‐15)	.534	76.5	1.00	14	.802
				ODP	34	85.3		8 (2‐22)		76.5		14	
Stauffer^89^	2016	Retr	USA	LDP	44	95.5	.1012	25.9 (5‐48)	.0001	75.6	1.00	3ys, 44%	.22
				ODP	28	82.8		12.7 (1‐45)		75.0		3ys, 41%	
Zhang^90^	2017	Retr	China	LDP	22	91.0	.61	11.2 ± 4.6	.44	‐	‐	29.6	.34
				ODP	76	87.0		14.4 ± 5.5		‐		27.6	
Kantor^91^	2017	Retr	USA	LDP	349	82.2	<.01	14.0 ± 11.7	.31	67.9	.05	29.9	.09
				ODP	1205	75.1		14.8 ± 12.0		61.8		24	
Bauman^92^	2018	Pros	USA	LDP	33	77	.53	14.5 ± 1.1	.07	61.0	.83	5ys, 20%	.39
				ODP	46	87		17.5 ± 1.2		63.0		5ys, 15%	
Raoof^93^	2018	PSM	USA (NCDB)	LDP	563	85.1	.11	12 (7‐18)	.759	‐	‐	HR 0.93, p = 0.457	
				ODP	563	81.5		12 (6‐18.5)		‐			
van Hilst^94^	2019	PSM	Europe	LDP	340	67.0	.019	14 (8‐22)	<.001	76.0	.561	28	.774
				ODP	340	58.0		22 (14‐31)		73.0		31	

Abbreviations: 3ys, three year survival; 5ys, five year survival; AT rate, induction rate of adjuvant treatment; FHD, French healthcare databases; HR, hazard ratio; LDP, laparoscopic distal pancreatectomy; LN, lymph node; mo, months; MST, median survival time; NCDB National Cancer Database; ODP, open distal pancreatectomy; Pros, prospective study; PSM, propensity score matching analysis; Retr, retrospective study.

### 3.2 Laparoscopic pancreaticoduodenectomy

Three RCTs comparing laparoscopic pancreaticoduodenectomy (LPD) **(**Table 3 **)** and open pancreaticoduodenectomy (OPD) have been reported to date.^76^, ^77^, ^78^ In all studies, although LPD was associated with a prolonged operative time, short‐term outcomes such as complication rates, mortality rates, and costs were equivalent between the two procedures. Two single‐center RCTs reported a short duration of hospital stays after LPD.^76^, ^77^ Conversely, in one multicenter RCT, the 90‐day mortality associated with LPD was as high as 10% (*P* = .2), although the complication rate was equivalent to that of OPD. Consequently, that RCT was terminated prematurely^78^. An oncological retrospective comparison of LPD and OPD for PDAC reported that the R0 resection rate, number of retrieved lymph nodes, MST (approximately 20 months), and 5‐year survival rate (20%‐30%) were equivalent between the procedures.^96^, ^97^, ^98^, ^99^, ^100^, ^101^ The oncological outcomes were also comparable in the three PSM analyses.^101^, ^102^, ^103^ In a recent meta‐analysis, a significantly higher R0 resection rate and a significantly higher number of lymph node dissections were reported for LPD; however, the 5‐year survival rate for LPD was equivalent to that of OPD.^104^ The postoperative mortality rate for LPD was higher in the low‐volume center than in the high‐volume center.^97^, ^99^, ^105^, ^106^ The complication rate was lower in the institution with MIPD >20 cases per year or PD >20 cases per year,^107^ and it was also reported that the mortality rate was lower in the institution with PD >10 cases per year.^97^, ^101^ Therefore, it is necessary to consolidate LPD patients into a high‐volume center for their safety as well as to provide appropriate educational guidance to surgeons and facilities.^11^, ^108^

**Table 3:** Comparison of oncological outcomes between MIPD and OPD for pancreatic cancer

Author	Year	Study design	Country	n		Mortality, %	*P*	R0 rate, %	*P*	Harvested LNs	*P*	MST, mo	*P*
Croome^96^	2014	Rter	USA	LPD	108	1.0	.5	77.8	.45	21.4 ± 8.1	.15	25.3	.12
				OPD	214	2.0		76.6		20.1 ± 7.5		21.8	
Sharpe^97^	2015	Rter	USA (NCDB)	LPD	384	5.2	.163	80	.001	18 ± 9.7	.0001	‐	‐
				OPD	4037	3.7		74		65 ± 9.6		‐	
Stauffer^98^	2017	Rter	USA	LPD	58	3.4	.737	84.5	.426	27 (9‐70)	<.001	5ys, 32.1%	.249
				OPD	193	5.2		79.8		17 (1‐63)		5ys, 15.3%	
Chapman^105^	2017	Rter	USA (NCDB)	LPD	248	4.9	.61	77.4	.12	>10, 69.0%	.57	19.8	.022
				OPD	1520	5.9		73.0		>10, 67.8%		15.6	
Kantor^99^	2017	Rter	USA (NCDB)	LPD	828	4.1	.71	79.1	.13	18.1 ± 9.5	.01	20.7	.68
				OPD	7385	3.8		76.8		17.1 ± 9.6		20.9	
Kuesters^100^	2018	Rter	Germany	LPD	62	4.8	.23	87	.01	16 (2‐47)	.69	5ys, 20.0%	.51
				OPD	278	2.2		71		17 (7‐28)		5ys, 14.0%	
Torphy^101^	2019	PSM	USA (NCDB)	MIPD	3753	5.0	.464	84.6	.133	>16, 48.1%	.305	‐	‐
				OPD	18259	6.7		80.0		>16, 45.2%		‐	
Zhou^102^	2019	PSM	China	LPD	55	0.0	.53	100	.201	18 (13‐25)	<.001	20	.293
				OPD	93	2.2		94.6		11 (7‐14.5)		18.7	
Kwon^103^	2020	PSM	Korea	MIPD	73	0.0	.589	75.0	.526	18.6 ± 9.9	.006	27.6	.079
				OPD	219	0.7		71.6		22.1 ± 10.6		24.5	

Abbreviations: 5ys, five year survival; LNs, lymph nodes; LPD, laparoscopic pancreaticoduodenectomy; MIPD, minimally invasive pancreaticoduodenectomy; mo, months; MST, median survival time; NCDB, National Cancer Database; OPD, open pancreaticoduodenectomy; PSM, propensity score matching analysis; R0 rate, R0 resection rate; Retr, retrospective study.

### 3.3 Robotic pancreatectomy

Robotic surgery provides a magnified view, and extremely sophisticated three‐dimensional images are associated with high operability **(**Table 4 **)**; therefore, robotic surgery is expected to overcome the limitations of laparoscopic surgery. However, a recent meta‐analysis^109^, ^110^ and PSM analysis^111^ have reported that the frequency of postoperative pancreatic fistula (POPF) and overall complication rates were equivalent between robotic and laparoscopic DP. In addition, a recent meta‐analysis comparing the perioperative outcomes of robotic and laparoscopic PD reported that the perioperative outcomes were similar between the two approaches.^71^, ^112^ Table 4 shows a comparison of the oncological outcomes between robotic pancreatic surgery and laparoscopic surgery, as well as between robotic and open surgery for PDAC.

**Table 4:** Comparison of oncologic outcomes among robotic, laparoscopic and open pancreatectomy for pancreatic cancer

Author	Year	Study design	n		Mortality, %	*P*	Oncological outcomes							
							R0 rate, %	*P*	Harvested LNs	*P*	AT rate, %	*P*	MST, mo	*P*
DP														
Raoof^93^	2018	Rter (NCDB)	RDP	99	0.0	.1	84	.84	11	.67	69.0	.82	3ys, 46%	.71
			LDP	605	3.0		85		12		59.0		3ys, 43%	
Girgis^113^	2019	Rter (NCDB)	RDP	48	6.25	1	93.75	.222	28.1	.304	80.0	.864	25.6	.055
			ODP	25	4.0		84.0		24.8		78.26		23.9	
Hong^114^	2019	Rter	RDP	12	0.0	‐	83.3	.621	17.9	.413	‐	‐	n.r.	.381
			LDP	76	0.0		89.5		15		‐		32.1	
Marino^115^	2020	CM	RDP	35	2.9	‐	100	.233	14.4	.678	‐	‐	3ys, 65.6	‐
			LDP	35	2.9		85		10.8		‐		3ys, 63.5	
Nassour^116^	2020	Rter (NCDB)	RDP	332	0.4	.002	85	.293	17	.002	‐	‐	35.3	<.001
			ODP	2386	4.4		81		15		‐		24.9	
PD														
Zureikat^117^	2016	Rter	RPD	70	1.9	.46	50	.002	27.5	<.001	‐		‐	‐
			OPD	452	2.82		69		19		‐		‐	
Nassour^118^	2018	Retr (NCDB)	RPD	147	4.8	.68	82.4	.289	18	.081	‐		22.7	.445
			LPD	165	5.6		79.6		17		‐		20.7	
Girgis^113^	2019	Retr (NCDB)	RPD	163	4.29	.908	78.53	.955	31.9	<.0001	67.9	.485	25.6	.055
			OPD	198	4.55		78.28		25.9		71.35		23.9	
Kauffmann^119^	2019	PSM	RPD	20	4.2	.34	55	.38	42	.2	75	.2	30.8	.87
			OPD	26	3.8		41.7		42		56.5		28.2	
Marino^121^	2019	CM	RPD	16	2.9	1.00	93.7	.023	26	.45	87.5	.22	65.2	.64
			OPD	13	2.9		76.9		21		84.6		62.3	
Baimas‐George^120^	2020	PSM	RPD	38	2.6	.5558	57.9	.817	21.5	.0036	68.4	n.s.	30.4	.1105
			OPD	38	5.3		55.3		13.5		68.4		23	
Nassour^116^	2020	Retr (NCDB)	RPD	626	4	.061	77	.052	22	<.001	‐	‐	22	.755
			OPD	17205	6		78		17		‐		21.9	

Abbreviations: 3ys, three year survival rate; AT rate, induction rate of adjuvant treatment; AT, adjuvant treatment; CM, case‐matched study; LPD, laparoscopic pancreaticoduodenectomy; mo, months; MST, median survival time; n.s., not significant.; NCDB, National Cancer Database; OPD, open pancreaticoduodenectomy; R0 rate, R0 resection rate; RDP, robot‐assisted distal pancreatectomy; Retr, retrospective analysis; RPD, robotic‐assisted pancreaticoduodenectomy.

In robot‐assisted distal pancreatectomy (RDP), the oncological outcomes of R0 resection rate and number of retrieved lymph nodes were comparable to those of laparoscopic and open DP.^93^, ^113^, ^114^, ^115^, ^116^

A study reported that the long‐term prognosis associated with RDP, however, was significantly better than that associated with open DP.^116^ In addition, the mortality rate and oncologic outcomes of robot‐assisted pancreaticoduodenectomy (RPD) were comparable to those of open surgery and laparoscopic surgery.^113^, ^116^, ^117^, ^118^, ^119^, ^120^, ^121^ In a meta‐analysis, the conversion rates of robotic PD and robotic DP were lower than those of laparoscopic surgery.^110^, ^112^ In particular, the lower emergency conversion rate is an advantage of robotic pancreatic surgery because lower emergency conversion is associated with many postoperative complications and patients that tend to present with poor prognoses.^110^, ^112^, ^122^

## 4 MULTIDISCIPLINARY TREATMENT

### 4.1 Postoperative adjuvant chemotherapy for resectable PDAC

Failure of the aggressive approach with extended lymph node **(**Table 5 **)** dissection to improve survival rate^123^, ^124^, ^125^, ^126^, ^127^ and the subsequent development of effective chemotherapy^128^, ^129^, ^130^, ^131^ has changed the standard treatment for R‐PDAC to R0 resection of the primary lesion and postoperative adjuvant chemotherapy.^132^, ^133^, ^134^, ^135^ Since 2017, three multi‐institutional RCT (ESPAC‐4, CONKO‐005, and PRODIGE) results have been published.^136^, ^137^, ^138^ In the ESPAC‐4 trial,^136^ the gemcitabine (GEM) plus capecitabine group had significantly better MST than that of the GEM alone group. In the PRODIGE study,^138^ comparing modified FOLFIRINOX (mFOLFIRINOX) and GEM, the median disease‐free survival (DFS) was 21.6 vs 12.8 months (HR 0.58; 95% confidence interval [CI], 0.46‐0.73; *P* < .001) and MST 54.4 vs 35.0 months (HR 0.64; 95% CI, 0.48–0.86; *P* = .003) were reported. The efficacy of mFOLFIRINOX for adjuvant chemotherapy was demonstrated. In the mFOLFIRIOX group, grade 3/4 adverse events occurred in 75.9% of the patients, but there was no mortality. Furthermore, the completion rate was 66.4%. Recently, preliminary results of gemcitabine plus nab‐paclitaxel (APACT study)^139^ as adjuvant chemotherapy were reported at the ASCO 2019 annual meeting. The prolongation of MST was shown to be 40.6 vs 35.2 months, *P* = .045; more conclusive results are eagerly awaited.

**Table 5:** Clinical trials on adjuvant chemotherapy for pancreatic cancer

Author	Year	Study	Design	n	mDFS, mo	*P*	MST, mo	*P*
Oettle^132^	2013	CONKO‐001	GEM vs Surgery	354	13.4 vs 6.7	<.001	22.8 vs 20.2	.06
Neptolemos^134^	2010	ESPAC‐3	5‐FU/FA vs Surgery	458	‐	‐	23.2 vs 16.8	.003
Uesaka^135^	2016	JASPAC01	S‐1 vs GEM	385	22.9 vs 11.3	<.0001	46.5 vs 25.5	<.0001
Neptolemos^136^	2017	ESPAC‐4	GEM + Cap vs GEM	730	13.9 vs 13.1	.082	28.0 vs 25.5	.032
Sinn^137^	2017	CONKO‐005	GEM + Erulotinib vs GEM	436	11.4 vs 11.4	.26	24.5 vs 26.5	.61
Conroy^138^	2018	PRODIGE 24/CCTG PA.6	FOLFIRINOX vs GEM	493	21.0 vs 12.8	<.001	54.4 vs 35.0	.003

Abbreviations: Cap, capecitabine; FA, folinic acid; FOLFIRINOX, levofolinate + 5‐FU + irinotecan+oxaliplatin; GEM, gemcitabine; mDFS, median disease‐free survival; mo, months; MST, median overall survival.

### 4.2 Neoadjuvant therapy for R/BR‐PDAC

Although postoperative adjuvant chemotherapy has been effective, the actual rate of completion of courses of therapy has been limited due to postoperative complications and early recurrence after radical resection.^140^

Therefore, practitioners have started to conduct preoperative adjuvant treatment for controlling potential distant metastasis, improving local curativeness, and avoiding unnecessary surgery by excluding cases with aggressive tumors.^14^

#### 4.2.1 R‐PDAC

Few studies have demonstrated the efficacy of NAT for R‐PDAC. In a retrospective study of PDAC resection using the National Cancer Database (NCDB), MST was found to be significantly longer in neoadjuvant chemotherapy (NAC) than in adjuvant or surgery‐alone cases.^141^ Another retrospective study for stage I PDAC also reported that NAC had a high R0 resection rate and a favorable prognosis.^142^ PSM analysis using stage I/II resection cases from the NCDB reported improvement in MST in the NAT group (26 vs 21 months, *P* = .01).^142^ However, it must be noted that this trial had immortal time bias.^143^ In PSM analysis for patients with resected PDAC, survival times for NAT and that for upfront surgery (UpS) were equivalent in stage I (NAT vs UpS, 26.2 vs 25.7 months; *P* = .4418) and II patients (23.5 vs 23.0 months; *P* = .7751). However, in stage III patients, MST was significantly prolonged in the NAT group. (22.9 vs 17.3 months, *P* < .0001).^144^ In this way, there was a divergence in the results of PSM; therefore, the effectiveness of NAT for R‐PDAC patients has not yet been integrated into the equation.

In the PSM analysis of a single‐center, in which NAC was compared with neoadjuvant chemoradiotherapy (NACRT), NACRT had significantly better rates of negative resection margin (91% vs 79%, *P* < .01), negative lymph node metastases (53% vs 23%, *P* < .01), and local recurrence (16% vs 33%, *P* < .01). However, MST was reported to be comparable between the NAC and NACRT groups (33.6 vs 26.4 months, *P* = .09).^145^

Several meta‐analyses have been reported for NAT for R‐PDAC.^39^, ^69^, ^146^, ^147^, ^148^, ^149^, ^150^, ^151^, ^152^, ^153^, ^154^ The effectiveness of NAT in terms of OS improvement for R‐PDAC has not been clarified. In RCTs on NAC for R‐PDAC, only preliminary results have been reported. At the ASCO annual meeting in 2018, the results of a phase‐III clinical trial (PREPAC‐1) comparing NACRT and UpS for R/BR‐PDAC revealed that MST was significantly better in the NACRT group (13.5 vs 17.1 months; HR 0.71; *P* = .047).^155^ At the ASCO‐GI meeting in 2019, results of a Japanese RCT comparing NAC‐ GEM/S‐1 and UpS for R/BR‐PV PDAC were reported. The preoperative GEM/S‐1 group had significantly better MST (36.7 vs 26.6 months, HR 0.72, *P* = .015) than that of the UpS group.^149^, ^156^, ^157^ Some RCTs have included BR‐PDAC; therefore, the effectiveness of NAT for R‐PDAC has not yet been established. Currently, RCTs for NAT using GEM/Oxaliplatin^158^, ^159^, ^160^ and FOLFIRINOX for R‐PDAC are in progress. Conclusive results from these trials are awaited.

#### 4.2.2 Borderline resectable PDAC

Recently, it has been reported that NAT contributed to improved R0 resection rates and extended survival of BR‐PDAC patients.^161^ In a multicenter retrospective analysis in Japan, it was reported that the MST prolongation effect of NAT surpassed upfront surgery (25.7 vs 19.0 months; *P* = .015). However, there was no significant difference in survival time between neoadjuvant chemotherapy (NAC) and neoadjuvant chemoradiotherapy (NACRT) (MST, 29.2 vs 22.5 months; *P* = .130).^162^ A multicenter retrospective analysis of NAC using FOLFIRINOX and nano albumin bound‐paclitaxel (nab‐PTX) with gemcitabine (GEM) for BR/ LA‐PDAC showed a significant prolongation of MST in patients responding to chemotherapy.^163^

In addition, many single‐center retrospective analyses have reported the prolongation effect of MST on NAT.^29^, ^164^, ^165^, ^166^, ^167^, ^168^, ^169^ Two RCTs have recently been reported, which compared the efficacies of NAT and upfront surgery for BR‐PDAC. Jang et al conducted an RCT for BR‐PDAC, which compared a NAT group that underwent surgery after GEM‐based CRT where the surgery group underwent postoperative CRT. According to their report, ITT analysis showed that the survival time of the NAT group was significantly prolonged (MST: 21 vs 11 months, *P* = .028).^170^

Versteijne et al conducted an RCT, which compared a GEM‐based NACRT group with upfront surgery group for R/BR‐PDAC, but no survival‐prolonging effect was noted in ITT analysis（MST: 16.0 vs 14.3 months, *P* = .096).^171^ However, in the NACRT group, the R0 resection rate was improved, and disease‐free survival (DFS) was prolonged. Furthermore, the local recurrence rate decreased. By contrast, a recent meta‐analysis on NAT in BR‐PDAC reported that NAT contributed to the prolongation of survival as per ITT analysis (Table 6).^69^, ^146^, ^147^, ^148^, ^149^, ^150^, ^153^ Accordingly, there is sufficient evidence for the effectiveness of NAT for BR‐PDAC.

**Table 6:** Meta‐analyses of neoadjuvant therapy for BR pancreatic cancer

Author	Year	Number of study (Study design)	Period	n	NAT			UpS
					RR, %	R0 rate, %	MST, mo	MST, mo
Tang^146^	2016	18 (2 Pros/16 Retr)	1999‐2014	959	65.3	57.4	25.9	11.9
Zhang^147^	2017	39 (39 Pros)	2005‐215	1458	40.2	79.4	16.2	
Versteijine^148^	2018	38 (3 RCTs/21 Pros/ 4 Retr)	2005‐2016	9621	65.0	88.6	19.2	12.8
Unno^149^	2019	6^a^ (2 RCT/4 Retr)	2011‐2018	‐	OS: HR 0.66 (0.50‐0.87), *P* = .003			
Janssen^150^	2019	24 (8 Pros/16 Retr)	2012‐2017	313	67.8	83.9	22.2	
Pan^153^	2020	17^a^ (3 RCT/5 Pros/9 Retr)	2011‐2018	2286	OR 0.69 (0.41‐1.16), *P* = .159	OR 4.75 (2.85‐7.92), *P* < .001	HR 0.49 (0.37‐0.65), *P* < .001	
Cloyd^69^	2020	6^a^ (6 RCT)	2015‐2020	850	Risk ratio 0.93, (0.82‐1.04)	Risk ratio 1.51, (1.18‐1.93)	HR 0.73 (0.61‐0.86)	

Abbreviations: BR, borderline resectable pancreatic cancer; HR, hazard ratio.; mo, months; MST, median overall survival; NAT, neoadjuvant therapy; OR, odds ratio; OS, overall survival; Pros, prospective study; RCT, randomized controlled trial; Retr, retrospective study; RR, resection rate; UpS, upfront surgery.

^a^ Including studies for potentially resectable pancreatic cancer.

### 4.3 Conversion surgery for initially unresectable PDAC

Overall, in 70%–80% of all PDAC patients are diagnosed as “unresectable” (UR) at the first consultation due to locally advanced state (UR‐LA) or distant metastasis (UR‐M). The recent development of chemotherapeutic agents such as FOLFIRINOX^130^ and GEM/nabPTX,^173^ which have a high response rate in PDAC, and total neoadjuvant therapy (TNT) followed by continuous NACRT have reduced UR‐PDAC to R/BR‐PDAC. It is reported that, with primary excision after such potent NAT, a good long‐term prognosis is expected. In addition, conversion surgery has been reported to improve the prognosis of PDAC with regard to distant metastases.

The mortality rates of these conversion surgeries have been reported to be 0%‐7%, and complication rates have been reported to be 14%‐89%. Therefore, conversion surgery has been performed at an acceptable risk for selected patients.^35^ However, most of the reports of conversion surgery for unresectable PDAC were single‐center retrospective studies; therefore, the evidence of efficacy is limited.

Table 7 shows the results of a recent conversion surgery (Table 7). The median resection rate was 28.6% (range, 8%‐69%), the negative margin resection rate was 78.3% (range, 35%‐100%), and the MST was 12‐96 months for LA‐PDAC.^26^, ^29^, ^37^, ^174^, ^175^, ^176^, ^177^, ^178^, ^179^, ^180^, ^181^, ^182^, ^183^, ^184^, ^185^, ^186^, ^187^, ^188^, ^189^, ^190^, ^191^, ^192^, ^193^ The median resection rate was 14.3% (range, 2%‐43%), the margin‐negative resection rate was 88% (range, 51%‐91.3%), and the MST was 21.9‐56 months, even in advanced PDAC with distant metastases.^34^, ^194^, ^195^, ^196^, ^197^, ^198^

**Table 7:** Reports of conversion surgery for unresectable pancreatic cancer

Author	Year	Study design	n	Resectability	Treatment regimen	Conversion			Not resected	*P*
						RR, %	Margin‐negative rate, %	MST, mo	MST, mo	
Nanda^181^	2015	Retr	29	BR/LA	FFX + SBRT	41.3	83.0	‐	‐	
Reni^184^	2017	Retr	223	BR/LA	GEM based	27.0	‐	30	16.5	<.00001
Veldhuisen^37^	2018	Retr	54	BR/LA	FFX	20.3	55	29	16	.02
Maggino^192^	2019	Pros	680	BR/LA	FFX/GnP/others	15.1	57.8	41.8	‐	
Yoo^189^	2019	Retr	135	BR/LA	FFX/GEM based	‐	76	29.7	‐	
Michelokos^29^	2019	Retr	141	BR/LA	FFX	‐	80.6	37.7	18.6	<.01
Rangelova^190^	2019	Retr	156	BR/LA	FFX/others/CRT	33.3	‐	22.4	12.7	<.0001
Byun^198^	2019	Retr	337	BR/LA/M	FFX	18.0	7	21	‐	
Bickenbach^174^	2012	Pros	‐	LA	‐	‐	83	30	‐	
Strobel^175^	2012	Pros	257	LA	CT/CRT	47	35	12.7	8.8	<.0001
Herman^178^	2015	Pros	49	LA	GEM + RT	8.0	100	22.2	13.8	.182
Marthey^180^	2015	Pros	77	LA	FFX	36.0	89	24.9	‐	
Sherman^182^	2015	Pros	45	LA	GTX/GX + CRT	64.4	69	‐	‐	
Sadot^179^	2015	Retr	101	LA	FFX	31.0	55	n.r.	‐	
Bednar^183^	2017	Retr	92	LA	FFX/GnP/others	20.0	‐	32	14.3	.0002
Lee^188^	2018	Retr	64	LA	FFX	23.0	73.3	n.r.	13	
Gemenetzis^187^	2019	Retr	415	LA	FFX based/GEM based/others	20.0	89.0	35.3	16.2	.001
Murphy^191^	2019	Pros	49	LA	FFX + Losartan	69.0	88	31.4	‐	
Napolitano^193^	2019	Retr	56	LA	FFX	40.0	1.43	96.0	72.1	.0006
					GnP	28.6	83.3	62.6	53.3	.0166)
Satoi^176^	2013	Retr	159	LA/M	Multi	‐	‐	39.7	20.8	<.0001
Opendro^177^	2014	Retr	130	LA/M	Multi	10.0	84.6	36	9	<.001
Hackert^26^	2016	Pros	575	LA/M	FFX/GEM + RT/others	50.8	75.0	15.3	8.5	<.0001
Asano^185^	2018	Retr	‐	LA/M	Multi	‐	88.2	63	‐	
Heger^32^	2019	Retr	318	LA/M	FFX	52.0	‐	23	‐	
Natsume^186^	2019	Retr	434	LA/M	FFX/GnP	4.1	88.9	n.r.	11	<.001
Klaiber^33^	2019	Retr	‐	LA/M	FFX/GEM based/others	‐	64.6	25.1	‐	
Crippa^194^	2016	Retr	127	M	Multi	8.7	82	39	11	<.0001
Wright^195^	2016	Retr	1147	M	FFX	2.0	91.3	34.1	‐	
Satoi^196^	2017	Pros	33	M	S‐1 + PTX (iv + ip)	24.0	‐	26	14.2	.0038
Frigerio^197^	2017	Retr	535	M	FFX	4.5	88	56	‐	
Tanaka^34^	2019	Retr	101	M	FFX	43.0	51	21.9	16.4	.006

Abbreviations: BR, borderline resectable pancreatic cancer; CRT, chemoradiotherapy; CT, chemotherapy; FFX, FOLFIRINOX; GEM, gemcitabine; GnP, gemcitabine + nab‐paclitaxel; GTX, gemcitabine + docetaxel; GX, gemcitabine + capecitabine; i.p., intraperitoneal infusion; i.v., intravenous infusion; LA, locally advanced unresectable pancreatic cancer; M, metastatic pancreatic cancer; mo, months; MST, median survival time; Multi, multiple regimen; Pros, prospective study; PTX, paclitaxel; Retr, retrospective study; RT, radiation; SBRT, stereotactic body radiotherapy; UR, unresectable pancreatic cancer.

Accordingly, even in UR‐LA and UR‐M PDACs, good prognosis was feasible when resection was performed, and MST was equivalent to that of R‐PDAC. However, there are no standard criteria for appropriate indication, optimal timing, and preoperative treatment regimen for conversion surgery. CA19‐9 level is the most effective biomarker for predicting the potential for resection. To avoid early recurrence after conversion surgery and to obtain a good long‐term prognosis, reduction or normalization of CA19‐9 levels after TNT is a necessary requirement（see 2.2 Biomarker‐based resectability criteria). Furthermore, negative FDG accumulation on PET, which is a metabolic biomarker, and a long period of chemotherapy are also advantageous for long‐term survival after conversion surgery. In the future, it is necessary to continue to investigate and determine the optimal criteria for conversion surgery.

## 5 CONCLUSION

We reviewed the recent trends in surgical treatment for PDAC and summarized the important points. Significant advances in surgical and multimodality treatments are increasing the range of options for treating PDAC. In the future, in order to steadily improve treatment results, not only is research on new biomarkers for assessing operability and tumor dynamics desirable, but research on the development of new anti‐cancer therapeutic agents and new multidisciplinary treatment methods is essential.

## Conflicts of Interest

Authors declare no conflicts of interest for this article.
